# Assessment of urban ecosystem health and its influencing factors: a case study of Zibo City, China

**DOI:** 10.1038/s41598-024-59103-6

**Published:** 2024-04-11

**Authors:** Xiaoming Wang, Qianqian Dong

**Affiliations:** College of Geography and Environment, Shandong Normal University, Ji’nan, 250358 Shandong China

**Keywords:** Urban ecosystem health, Obstacle factor analysis, Sensitivity factor analysis, Zibo City, Ecology, Environmental social sciences

## Abstract

Urban ecosystem health is the foundation of sustainable urban development. It is important to know the health status of urban ecosystem and its influencing factors for formulating scientific urban development planning. Taking Zibo city as the study area, the indicators were selected from five aspects: ecosystem vigor, structure, resilience, service function and population health to establish an assessment index system of urban ecosystem health. The health level of urban ecosystem was assessed, and its changing trend was analyzed from 2006 to 2018 in Zibo. Furthermore, obstacle degree analysis and sensitivity analysis were used to quantitatively analyze the main obstacle factors and sensitivity factors affecting urban ecosystem health, so as to provide references for improving urban ecosystem health. The results showed that the health level of urban ecosystem in Zibo showed an upward trend from 2006 to 2018. The poor structure and ecological environment quality were the main obstacle factors to urban ecosystem health. The impact of changes in a single indicator on urban ecosystem health gradually decreased, but the sensitivity index of indicators had obvious differences. Urban ecosystem health was sensitive to changes in ecosystem structure and resilience. In the future, Zibo should strengthen ecological construction, optimize the industrial structure, and develop green economy to promote urban ecosystem healthy.

## Introduction

Ecosystem provides material and energy for human survival and development, and is also affected by human activities. Since Rapport put forward the concept of ecosystem health in 1989^1^, ecosystems and their health have attracted increasing attention. Urban ecosystem is a highly artificial natural, social and economic complex ecosystem. From the perspective of the relationships among nature, economy and society, urban ecosystem health not only refers to the health of the natural environment, but also includes the healthy urban economy and human health. A healthy urban ecosystem should have a reasonable structure, perfect functions, and be able to continuously provide the required material output and ecological services for urban residents, and prevent damage to residents' health. Urban ecosystem is more affected by human activities than natural ecosystem. With the process of urbanization, the impact of population aggregation and the increasing intensity of socio-economic activities on the urban ecosystem is growing, which has resulted in many social, economic and eco-environment problems^2,3^. Urban ecosystem health is an important guarantee for urban development, so maintaining urban ecosystem health to promote sustainable urban development has become an important content of urban planning and urban ecology research^4–7^. Recently, the research on urban ecosystem health mainly involves the concept and connotation, assessment index system, assessment methods and empirical research^8–11^.

Establishing an index system is the key point of urban ecosystem health assessment. Due to complexity of urban ecosystem, various index systems have been constructed from different perspectives, such as vigor-structure-resilience framework^12,13^, vigor-organization-resilience framework^14^, structure–function–process–development framework^15^, natural-social-economic framework^16,17^, pressure-state-response framework^18,19^, ecosystem services-landscape patterns framework^20^, and infrastructure-economy-ecology-society framework^21^. These frameworks reflect the characteristics of urban ecosystem and the relationship between humans and the environment in different aspects, but to some extent they are not enough to reveal the complexity of urban ecosystem and the essence of health. As a highly artificial natural, social and economic complex ecosystem, ecosystem’s service function and public health should also be considered in the assessment of urban ecosystem health. On the basis of the vigor-structure-resilience framework, the vigor-structure-resilience-function-population health framework was proposed^22,23^, which further improved the assessment index system of urban ecosystem health. At present, many mathematical models have been applied to assess ecosystem health, such as element analysis^22^, energy analysis^24,25^, fuzzy matter-element extension model^26,27^, comprehensive assessment model^28^, set pair analysis^29^, material flow analysis^30^, maximum information entropy method^31^, improved SI-MI model^32^, and variable fuzzy optimization model^33^. Although these methods play an important role in promoting the assessment of urban ecosystem health, many issues need further study. The highly artificial urban ecosystem has weak self-regulating ability, and ecosystem balance must be adjusted by human activities. Therefore, with targeted solutions to weak links in urban development, the research of urban ecosystem health needs not only to assess urban ecosystem health status, but also to analyze the reasons that affect its health. Studies have shown that urban expansion and urban form^7,34,35^, urban development policies^26^, ecological protection policies^36,37^, human capital^26,28^, geological environment, and socio-economic structure^36,38^ have potential impacts on urban ecosystem, but due to regional differences, the influencing factors of ecosystem health vary across cities. Most studies adopt qualitative analysis and comparative analysis methods. Some studies used quantitative methods to analyze the influencing factors of urban ecosystem health, such as fitting function^39^, boosted regression tree model^40^, artificial neural network model^41^, GTWR model^42^, and coupling coordination model^43^. These research methods can identify the main influencing factors of urban ecosystem health in a certain period, but they cannot reflect the change trend in the influence degree of factors, nor can they accurately reveal the obstacle factors and sensitivity factors that affect the healthy development of urban ecosystem. From the existing research, due to the complexity of urban ecosystem and the difference of urban development, a unified index system has not been formed. Many studies focused on ecosystem health characteristics, classification of health levels, and analysis of urban ecosystem health changes and regional differences^36,44^, but the research on in-depth analysis of the influencing factors of urban ecosystem health was relatively weak. Previous studies mainly focused on the health of urban ecosystem in large cities^11,23,40,43^, urban agglomerations^26,42^ and economically developed areas^22^, but paid less attention to the ecosystem health of typical industrial cities. Especially since the policy of energy conservation and emission reduction was proposed in the “11th Five-Year Plan” (2006–2010), under the background of continuously strengthening urban ecological construction, what is the health status of urban ecosystem in industrial city? What is the change trend? How to judge the influencing factors of urban ecosystem health? Research on these problems is helpful for managers to take effective measures to promote urban ecosystem healthy.

Taking Zibo city as the study area, according to the characteristics of industrial cities, this paper selected assessment indicators based on the vigor-structure-resilience-function-population health framework to construct the assessment index system, and assessed urban ecosystem health of Zibo city from 2006 to 2018. Then, obstacle degree analysis and sensitivity analysis were used to analyze the influencing factors of urban ecosystem health, which could not only calculate the impact of each factor on urban ecosystem health, but also find the obstacle factors and sensitivity factors affecting urban ecosystem health. This is helpful for city managers to take targeted measures to strengthen the sustainable management of urban ecosystem. Overall, this study provides a scientific decision-making reference for sustainable urban development in Zibo.

### Study area and data sources

Zibo city lies between 117° 32′–118° 31′ E and 35° 55′–37° 17′ N, with a total area of 5965 km^2^. In terms of topography, mountains are distributed in the east, south and west of the region, and the central part is lower and northward-inclined. The terrain is high in the south and low in the north, and the height difference between north and south is more than 1000 m. Centrally located in Shandong province, Zibo is not only an important transportation hub in the province with developed transportation, but also an important petrochemical, pharmaceutical production base and building materials production area in China with rich mineral resources. Due to its advantages of location and resource base, Zibo has become an important industrial city. However, the regional ecosystem health is facing the adverse effects of traditional industrial production models and industrial structures. Therefore, the urgent task for researchers and policymakers is to accelerate the transformation of urban economies and promote urban ecosystem health.

Taking Zibo city, Shandong province as the study area, the indicator data were collected from the municipal districts of Zibo. These data were sourced from China City Statistical Yearbook, Shandong Statistical Yearbook, Zibo Statistical Yearbook and Zibo Statistical Bulletin on National Economic and Social Development from 2007 to 2019. Among them, 7 indicators were obtained from China City Statistical Yearbook, including Per capita GDP, GDP growth rate, proportion of tertiary industry in GDP, urban sewage treatment rate, harmless treatment rate of domestic waste, comprehensive utilization rate of industrial solid waste and urban road area per capita, energy consumption per GDP and the proportion of environmental protection investment in GDP were retrieved from Shandong Statistical Yearbook, days with air quality of grade II and above was extracted from Zibo Statistical Bulletin on National Economic and Social Development, and the remaining 10 indictors were sourced from Zibo Statistical Yearbook.

## Methods

### Establishing the assessment index system

According to the meaning of urban ecosystem health, the vigor-structure-resilience-function-population health framework was adopted to construct the assessment index system in this study, which has been well applied in relevant research^26,31^. From previous research^11,22,23,45,46^, it can be seen that due to the differences in natural conditions, economic development level, industrial structure and development goals of different cities, although the selected evaluation indicators are different, this evaluation framework can reflect the productivity, structural complexity, stability, ability to provide services and maintain human health in urban ecosystem. We first sorted out and analyzed the evaluation indicators in the existing literature, and preliminarily selected some evaluation indicators. Then, according to the development characteristics of Zibo city, the preliminarily selected indicators were further analyzed and screened.

Life expectancy and under 5 mortality rate were selected as indicators of population health in previous studies. Since under 5 mortality rate data were incomplete in this study period and it only reflected the health status of children, and it is difficult to obtain data on life expectancy, we selected the mortality rate to reflect the overall health status of urban residents. From the existing indicator system, it can be found that there was a lack of indicators that reflect the medical health level of urban residents. In this study, health care expenditure of urban residents was added to reflect the ability of urban ecosystem to maintain human health. In addition, considering the characteristics of industrial city and the actual situation of Zibo city, some indicators were appropriately added. Days with air quality of grade II and above was selected to reflect air quality, proportion of high-tech industries in GDP was selected to reflect the industrial structure, two indicators, harmless treatment rate of domestic waste and proportion of environmental protection investment in GDP were selected to reflect the self-regulation ability of urban ecosystem under human management activities. Finally, evaluation indicators were selected from five aspects: ecosystem vigor, structure, resilience, service function and population health to construct the assessment index system of urban ecosystem health in Zibo (Table 1). (a) Vigor, which reflects a city's economic vitality and green production efficiency. We selected three indicators: Per capital GDP (*x*_1_), GDP growth rate (*x*_2_) and Energy consumptions per GDP (*x*_3_). (b) Structure, which reveals the natural, economic and social structure in urban areas. We selected five indicators: Proportion of tertiary industry in GDP (*x*_4_), Proportion of high-tech industries in GDP (*x*_5_), Registered urban unemployment rate (*x*_6_), Urban population density (*x*_7_) and Green coverage rate in built-up area (*x*_8_). (c) Resilience, which reflects the self-regulating capacity of urban ecosystem. Since urban ecosystem is a highly artificial ecosystem, its self-regulating capacity is closely related to human management activities. Thus, Urban sewage treatment rate (*x*_9_), Comprehensive utilization rate of industrial solid waste (*x*_10_), Harmless treatment rate of domestic waste (*x*_11_), and Proportion of environmental protection investment in GDP (*x*_12_) were selected. (d) Service function, which reflects the ability of urban ecosystem to provide production and living services for urban residents. Per capita housing construction area (*x*_13_), Urban road area per capita (*x*_14_), Number of hospital beds per 10,000 people (*x*_15_), Days with air quality of grade II and above (*x*_16_), and Park green space per capita (*x*_17_) were selected. (e) Population health, which reflects the people-oriented characteristics of urban ecosystem. Engel’s coefficient of urban residents (*x*_18_), mortality rate (*x*_19_), and Health care expenditure of urban residents (*x*_20_) were selected to reflect the living standard and health status of urban residents.

**Table 1:** Assessment index system of urban ecosystem health in Zibo.

Assessment factor	Factor weight	Assessment indictor	Indictor weight	Indictor feature (ideal value)
Vigor	0.149	Per capital GDP (10,000 yuan/person)* x*_1_	0.353	Positive
		GDP growth rate (%) *x*_2_	0.271	Appropriate (10)
		Energy consumptions per GDP (t (standard coal)/10,000 yuan) *x*_3_	0.376	Negative
Structure	0.271	Proportion of tertiary industry in GDP (%) *x*_4_	0.24	Positive
		Proportion of high-tech industries in GDP (%) *x*_5_	0.275	Positive
		Registered urban unemployment rate (%) *x*_6_	0.25	Negative
		Urban population density (person/ km^2^) *x*_7_	0.119	Appropriate (10,000)
		Green coverage rate in built-up area (%) *x*_8_	0.116	Appropriate (50)
Resilience	0.201	Urban sewage treatment rate (%) x_9_	0.251	Positive
		Comprehensive utilization rate of industrial solid waste (%) *x*_10_	0.197	Positive
		Harmless treatment rate of domestic waste (%) *x*_11_	0.187	Positive
		Proportion of environmental protection investment in GDP (%) *x*_12_	0.365	Positive
Service function	0.165	Per capita housing construction area (m^2^/person) *x*_13_	0.169	Appropriate (40)
		Urban road area per capita (m^2^/person) *x*_14_	0.218	Appropriate (20)
		Number of hospital beds per 10,000 people (bed) *x*_15_	0.175	Appropriate (70)
		Days with air quality of grade II and above (%) *x*_16_	0.264	Positive
		Park green space per capita (m^2^/person) *x*_17_	0.174	Appropriate (20)
Population health	0.214	Engel’s coefficient of urban residents (%) *x*_18_	0.399	Negative
		mortality rate (‰) *x*_19_	0.179	Negative
		Health care expenditure of urban residents (yuan/person) *x*_20_	0.422	Positive

### Calculating the weight of each indictor

#### Data standardization

In order to eliminate the influence of different units, features, and orders of magnitude of indicators, the original data should be firstly standardized. According to the relationship between the indicator and the evaluation goal, the indicator can be divided into three types, positive indicators, negative indicators and appropriate indicators. Positive indicator suggests that a higher value indicates a healthier urban ecosystem, while negative indicator suggests the opposite. Appropriate indicator means that only when the indicator reaches a specific optimal level does it contribute to urban ecosystem health. Among the appropriate indicators, GDP growth rate reflects the speed of urban economic development. From the perspective of sustainable development, rapid GDP growth is not always optimal, and moderate growth is more conducive to the healthy development of cities. During the period of “11th Five-Year Plan” (2006–2010), GDP grew by 14.21% annually in Zibo, higher than the national average. Since 2011, the strategy of sustainable development has been carried out, which no longer pursuing rapid growth in the economic quantity, but paying more attention to the quality of economic growth. Combining with the “12th Five-Year Plan” (2011–2015) and “13th Five-Year” Plan (2016–2020) of Zibo city, 10% is taken as the ideal GDP growth rate. The scale effect brought by urban population agglomeration is beneficial to urban development, but excessive population density will also lead to negative effects such as “urban disease”. Therefore, urban population density should be controlled reasonably. In China, urban land area is allocated according to urban population in urban development planning^47^. According to the standard of 100 m^2^ per capita, the urban population density should reach 10,000 people per square kilometer. In addition, considering the scarcity of land resources and environmental protection, the ideal values of appropriate indicators are determined by referring to the recommended values of ecological city, healthy city, garden city and environmental protection city at home and abroad to improve the utilization efficiency of public resources and rationally adjust land use structure (Table 1).

The values of various indicators are standardized to make them between 0 and 1. The formula is as follows:

1 $${x}_{ij}{\prime}=\frac{{x}_{ij}-{x}_{jmin}}{{x}_{jmax}-{x}_{jmin}}\text{positive indicator}$$

2 $${x}_{ij}{\prime}=\frac{{x}_{jmax}-{x}_{ij}}{{x}_{jmax}-{x}_{jmin}}\text{negative indicator}$$

3 $${x}_{ij}{\prime}=1-\frac{\left|{x}_{ij}-{x}_{j}^{*}\right|}{{x}_{j}^{*}}\text{ appropriate indicator}$$

where *x*_*ij*_ is the original value of indictor *j* in year *i*; *x*^*’*^_*ij*_ is the normalized value of indictor *j* in year *i*; *x*_*jmax*_ is the maximum value of indictor* j* in the study period; *x*_*jmin*_ is the minimum value of indictor *j* in the study period; and x^*^_j_ is the ideal value of indictor *j.*

#### Weight calculation

The entropy weight method is used to calculate the weight of each indictor, and the formula is as follows:

Calculating the proportion of indictor *j*, *R*_*ij*_

4 $$R_{{ij}} = x_{{ij}} \prime /\sum\limits_{{i = 1}}^{{\text{m}}} {x_{{ij}} \prime }$$

Calculating the entropy of indictor *j*, *e*_*j*_

5 $$e_{j} = - \left( {\frac{1}{{{\text{l}}\;{\text{nm}}}}} \right)\sum\limits_{{i = 1}}^{m} {R_{{ij}} {\text{ln}}R_{{ij}} }$$

Calculating the difference coefficient of indictor *j*, *g*_*j*_

6 $${g}_{j}=1-{e}_{j}$$

Calculating the weight of indictor *j*, *w*_*j*_

7 $${w}_{j}=\frac{{g}_{j}}{{\sum }_{j=1}^{n}{g}_{j}}=\frac{1-{e}_{j}}{{\sum }_{j=1}^{n}1-{e}_{j}}$$

where *x*^*’*^_*ij*_ is the normalized value of indictor *j* in year *i*; *m* is the number of the assessed years; and *n* is the number of indicators.

Since the entropy weight method uses the concepts of entropy and logarithm, subject to relevant rules, negative value cannot be involved in the calculation. Then, when *R*_*ij*_ = 0, 0.00001 is used instead. The calculation results of indictor weight are shown in Table 1.

### Urban ecosystem health assessment model

The multi-factor comprehensive evaluation model is adopted to calculate the urban ecosystem health index. The formula is as follows:

8 $$F=\sum_{i=1}^{m}{w}_{i}\sum_{j=1}^{n}({w}_{j}\cdot {x}_{ij}{\prime})$$

where *F* is the urban ecosystem health index; *x*^*’*^_*ij*_ is the normalized value of indictor *j*; *w*_*j*_ is the weight of indictor *j*; *w*_*i*_ is the weight of assessment factor *i*; *m* is the number of assessment factors; and *n* is the number of indicators in the assessment factor *i.*

### Rank correlation analysis method

Spearman rank correlation analysis is used to analyze the changing trend of urban ecosystem health index. The calculation can be expressed as:

9 $$r_{s} = 1 - \left[ {6\sum\limits_{{i = 1}}^{n} {d_{i}^{2} } } \right]/[N^{3} - N]$$

10 $${d}_{i}={x}_{i}-{y}_{i}$$

where *r*_*s*_ is the Spearman rank correlation coefficient; *x*_*i*_ and *y*_i_ are the serial number of urban ecosystem health index and year from small to large, respectively, namely rank order; *d*_*i*_ is the difference between *x*_*i*_ and* y*_*i*_; and *N* is the number of samples.

### Obstacle degree method

By introducing contribution index, deviation degree index, and obstacle degree index, the obstacle degree model is used to diagnose the obstacle factors that affect urban ecosystem health. The formula is as follows:

11 $${V}_{j}={w}_{j}\cdot {w}_{i}$$

12 $${P}_{j}=1-{x}_{j}{\prime}$$

13 $$A_{j} = P_{j} \cdot V_{j} /\sum\limits_{{j = 1}}^{n} {(P_{j} \cdot V_{j} )} \times 100\%$$

where* V*_*j*_ is the contribution degree of indictor *j*; *P*_*j*_ is the deviation degree of indictor *j*; *A*_*j*_ is the obstacle degree index of indictor *j*; *w*_*j*_ is the weight of indictor *j*; *w*_*i*_ is the weight of factor *i* to which indictor *j* belongs; *x*^*’*^_*j*_ is the normalized value of indictor *j*; and *n* is the number of indictors.

### Sensitivity analysis method

Referring to relevant research^48–50^, the elasticity analysis model is used to calculate the impact intensity of indictor changes on urban ecosystem health. The sensitivity index is defined as the ratio of the change rate of urban ecosystem health index to that of the indictor, that is, the change degree of urban ecosystem health index caused by 1% of the indictor change. The larger the sensitivity index, the more sensitive the urban ecosystem health index is to the change of indictor value, and the greater the impact of indicator change on urban ecosystem health. Conversely, the less sensitive it is, the smaller the impact of indicator change on urban ecosystem health. The formula is as follows:

14 $$CS=\left|\frac{({F}_{n}-{F}_{m})/{F}_{m}}{({x}_{n}-{x}_{m})/{x}_{m}}\right|$$

where *CS* is the sensitivity index; *F*_*m*_ and *F*_*n*_ are the initial index and the adjusted index of urban ecosystem health, respectively; *x*_*m*_ and *x*_*n*_ are the initial value and the adjusted value of indicator, respectively.

## Results

### Analysis of urban ecosystem health change in Zibo

#### Overall change characteristics of urban ecosystem health

On a whole, the health level of urban ecosystem in Zibo showed a fluctuating upward trend (Table 2). The health index of urban ecosystem increased from 0.329 in 2006 to 0.897 in 2018, which increased by 2.73 times.

**Table 2:** Health index of urban ecosystem in Zibo.

Year	2006	2007	2008	2009	2010	2011	2012	2013	2014	2015	2016	2017	2018
Vigor index	0.183	0.257	0.423	0.490	0.533	0.587	0.682	0.641	0.740	0.692	0.845	0.871	0.898
Structure index	0.227	0.189	0.180	0.292	0.377	0.351	0.581	0.532	0.562	0.583	0.683	0.691	0.901
Resilience index	0.209	0.242	0.203	0.523	0.620	0.687	0.762	0.750	0.825	0.911	0.954	0.848	0.896
Service function index	0.677	0.706	0.720	0.758	0.769	0.821	0.864	0.659	0.727	0.654	0.709	0.768	0.787
Population health index	0.404	0.279	0.203	0.342	0.323	0.434	0.251	0.297	0.318	0.833	0.886	0.770	0.975
Ecosystem health index	0.329	0.314	0.315	0.455	0.502	0.549	0.609	0.563	0.616	0.730	0.810	0.779	0.897

At different stages of socio-economic development, obvious differences emerged in the health status and its changes of urban ecosystem in Zibo. From 2006 to 2010, the urban ecosystem health index was low, with an average value of 0.383, but showed a rapid improvement, with an average annual increase of 13.2%, which was consistent with the development characteristics of Zibo. On the one hand, as an industrial city, Zibo had a high proportion of the secondary industry, but a relatively low percentage of high-tech industry, accounting for about 20% of the total output value of industries above designated size, resulting in higher energy consumption and waste output. Despite the rapid growth of GDP in this period, the comprehensive utilization rate of industrial solid waste and environmental protection investment were relatively low because of the weak technical level and weak environmental awareness, leading to the lower health level of urban ecosystem. On the other hand, guided by the policy of “energy conservation and emission reduction” put forward in the “11th Five-Year Plan” (2006–2010), the new industrial production concept was applied to gradually reduce energy consumption and improve the comprehensive utilization rate of waste. Simultaneously, with the gradual increase of environmental protection investment, the ecological environment improved significantly, and the health level of urban ecosystem continued to improve. The urban ecosystem health index increased by 52.76% from 2006 to 2010.

From 2011 to 2018, the health level of urban ecosystem kept improving, but the rate of improvement tended to be gentle. From 2011 to 2015, the average urban ecosystem health index was 0.613, with an average annual increase of 8.13%. During this period, under the guidance of the policies of “transformation of Old–New-Driving-Forces” and “energy conservation and emission reduction”, Zibo paid attention to urban ecological construction. Urban living environment was constantly improved by adjusting the industrial structure and increasing investment in environmental protection. Although the GDP growth showed a slowdown during the “12th Five-Year Plan” (2011–2015), the overall health level of urban ecosystem improved. From 2016 to 2018, the average urban ecosystem health index reached 0.828, with an annual increase of 7.39%, indicating that under the background of ecological civilization construction, Zibo no longer seeks economic development at the expense of ecological environment. In the early stage of “13th Five-Year Plan” (2016–2018), the health status of urban ecosystem reached a high level, and urban ecosystem was in a stable development state.

#### Change of subsystem health

From the perspective of five subsystems, vigor index, structure index, resilience index and population health index all changed greatly, while service function index fluctuated, but kept at a high level, indicating that Zibo paid attention to people's livelihood in urban construction and offered relatively perfect infrastructure and public service facilities.

From 2006 to 2010, vigor index and resilience index were both at a low level. Although economy developed rapidly during this period, due to the influence of traditional industrial production concepts and technologies, high energy consumption, insufficient investment in environmental protection and low comprehensive utilization rate of waste led to low vitality and resilience of urban ecosystem in Zibo. This reflected that the ecological environment problems caused by rapid economic development in this period were more prominent, and the development mode at the expense of the environment was not conducive to ecosystem healthy. After 2010, guided by the “energy conservation and emission reduction” policy and the concept of new industrial production, Zibo strengthened urban ecological construction. Through technological innovation and increased environmental protection input, energy consumption per GDP reduced by 6% annually, harmless treatment rate of domestic waste reached 100%, and urban sewage treatment rate and the comprehensive utilization rate of industrial solid waste increased by 10.46% and 7.37% respectively, thus enhancing the vitality and resilience of urban ecosystem.

The structure index showed a fluctuating upward trend. From 2006 to 2015, the structure index was low, which was manifested as the unreasonable industrial structure, low proportion of high-tech industry and the underdeveloped tertiary industry in Zibo. After 2015, with the implementation of the Old–New-Driving-Forces conversion policy, Zibo actively promoted the transformation and upgrading of old industries. By transforming old industrial enterprises, introducing high-tech industries and developing the tertiary industry, the industrial structure was constantly improved and the structure of urban ecosystem was also constantly optimized.

The population health index fluctuated greatly. Especially in 2012–2014, there was a significant decline, which may be due to the influence of policies. Since 2012, the GDP growth rate in Zibo began to decline, which affected residents' income and expenditure. In 2012, the Engel’s coefficient of urban residents increased by 4% compared with 2011, and the health care expenditure of urban residents also decreased correspondingly, by 14.54% compared with 2011. From 2015 to 2018, as the economy recovered and the medical security system continued to improve, the population health index continued to improve, and people's living conditions showed a positive trend.

#### The change trend of urban ecosystem health

When *N* = 13, critical value *W*_*P*_ = 0.56 (*P* < 0.05), and results of Spearman rank correlation analysis are showed in Table 3. The rank correlation coefficient of urban ecosystem health index is positive and higher than *w*_*p*_, indicating that the overall health level of Zibo’s urban ecosystem showed a significant improvement trend from 2006 to 2018. The variation trends of subsystem health are slightly different. The rank correlation coefficients of vigor index, structure index, resilience index and population health index are all positive and greater than *w*_*p*_, while the rank correlation coefficient of service function index is positive but less than *w*_*p*_. These results showed that the vigor, structure, resilience and population health of urban ecosystem in Zibo changed significantly and showed a significant improvement trend from 2006 to 2018, while the service function improved, but the improvement trend was not significant.

**Table 3:** Spearman rank correlation coefficient.

	Vigor index	Structure index	Resilience index	Service function index	Population health index	Ecosystem health index
Rank correlation coefficient (*r*_*s*_)	0.989	0.956	0.934	0.176	0.626	0.973

### Obstacle factor analysis

On the basis of calculating the factor obstacle degree for each year, the average value of factor obstacle degree of each stage was taken according to the stage characteristics of urban ecosystem health in Zibo. The factor obstacle degree was arranged from large to small, and the factors with a cumulative obstacle degree of more than 80% were regarded as the main obstacle factors affecting urban ecosystem health.

The main obstacle factors affecting urban ecosystem health in Zibo were different in different periods, and the number of factors showed a decreasing trend (Table 4). Compared with 2006–2010, the main obstacle factors decreased from 10 to 9 in 2011 to 2015, among which, the obstacle degree of urban sewage treatment rate (*x*_*9*_) and per capita GDP (*x*_*1*_) decreased significantly and were no longer the main obstacle factors, while the obstacle degree of days with air quality of grade II and above (*x*_*16*_) increased to be a new main obstacle factor, and the other eight main obstacle factors did not change. In general, during the period of “11th Five-Year Plan” (2006–2010) and “12th Five-year Plan” (2011–2015), the tertiary industry and high-tech industry accounted for a relatively low proportion in Zibo due to the influence of the industrial structure. In the process of rapid economic growth, high energy consumption in industrial production, insufficient investment in environmental protection and poor ecological environment were the constraints to urban ecosystem health. At the same time, the lack of expenditure on health care was also the main factor affecting population health in this period.

**Table 4:** Main obstacle factors of urban ecosystem health in Zibo, unit: %.

2006–2010		2011–2015		2016–2018	
Symbol	Obstacle degree	Symbol	Obstacle degree	Symbol	Obstacle degree
*x*_20_	13.54	*x*_20_	15.03	*x*_16_	19.63
*x*_5_	10.84	*x*_6_	11.30	*x*_7_	15.57
*x*_12_	9.70	*x*_18_	10.75	*x*_6_	14.98
*x*_4_	8.75	*x*_5_	8.79	*x*_2_	9.36
*x*_18_	8.48	*x*_12_	8.71	*x*_10_	7.53
*x*_6_	8.39	*x*_16_	7.36	*x*_20_	7.08
*x*_3_	7.20	*x*_7_	6.60	*x*_5_	5.88
*x*_1_	6.58	*x*_4_	6.28		
*x*_9_	5.24	*x*_3_	5.82		
*x*_7_	4.06				

From 2016 to 2018, the main obstacle factors were further reduced to seven, and the obstacle factors also changed significantly. With the implementation of policies to conserve energy, reduce emissions, and accelerate transformation of Old–New-Driving-Forces, in the early stage of “13th Five-Year Plan” (2016–2018), the industrial structure of Zibo was constantly optimized, economy developed steadily, and environmental protection investment was gradually increased. The obstacle degree of four factors: energy consumption per GDP (*x*_*3*_), the proportion of tertiary industry in GDP (*x*_*4*_), the proportion of environmental protection investment in GDP (*x*_*12*_) and Engel’s coefficient of urban residents (*x*_*18*_) were significantly reduced, which were no longer main obstacle factors. However, in the process of socio-economic transformation, the decline in economic growth affected the vitality of urban ecosystem, and GDP growth rate (*x*_*2*_) became the main obstacle factor. In addition, air quality, proportion of high-tech industry, comprehensive utilization of industrial solid waste, employment and health care of urban residents, and urban population density were still the main obstacle factors affecting urban ecosystem health.

It is worth noting that the proportion of high-tech industry in GDP (*x*_*5*_), registered urban unemployment rate (*x*_*6*_), urban population density (*x*_*7*_) and health care expenditure of urban residents (*x*_*20*_) are the main obstacle factors in each period. The proportion of high-tech industry in GDP (*x*_*5*_), registered urban unemployment rate (*x*_*6*_), and urban population density (*x*_*7*_) are all indicators reflecting the structure of urban ecosystem, indicating that poor structure is the main reason restricting urban ecosystem health in Zibo. Therefore, in the future, Zibo should continue to optimize the industrial structure, attract talents, increase employment rate, and improve residents' medical security.

### Sensitivity analysis

In 2006, 2010, 2015 and 2018, the values of each indicator were adjusted up (or down) by 1% respectively while keeping the values of other indicators unchanged to analyze the change of urban ecosystem health index caused by 1% change of indictor value (Table 5). The results showed that only the sensitivity index of the proportion of environmental protection investment in GDP (*x*_*12*_) increased from 2006 to 2018, whereas the sensitivity indexes of other indicators showed a decreasing trend, indicating that increasing environmental protection input had an important impact on improving urban ecosystem health in Zibo. Meanwhile, it also showed that before 2010, when the indicators were at a low level, improving the quality of a single indictor could effectively improve the health status of urban ecosystem. Conversely, a decline in the quality of an indicator would also lead to a decline in urban ecosystem health. After 2010, with the continuous improvement of the structure and function of urban ecosystem, the impact of changes in a single indictor on ecosystem health was getting weaker and weaker, reflecting the improvement of self-regulation and anti-interference ability of urban ecosystem, and the continuous improvement of urban ecosystem health in Zibo.

**Table 5:** Sensitivity index.

Year	Symbol									
	*x*_1_	*x*_2_	*x*_3_	*x*_4_	*x*_5_	*x*_6_	*x*_7_	*x*_8_	*x*_9_	*x*_10_
2006	0.108	0.163	0.332	0.407	0.345	1.113	0.024	0.076	0.845	0.657
2010	0.116	0.091	0.164	0.313	0.262	0.728	0.013	0.053	0.600	0.475
2015	0.100	0.032	0.092	0.259	0.226	0.520	0.011	0.039	0.430	0.346
2018	0.097	0.028	0.059	0.216	0.204	0.363	0.009	0.032	0.333	0.262

Year	*x*_11_	*x*_12_	*x*_13_	*x*_14_	*x*_15_	*x*_16_	*x*_17_	*x*_18_	*x*_19_	*x*_20_
2006	0.831	0.046	0.056	0.055	0.054	0.223	0.054	1.071	0.094	0.184
2010	0.559	0.087	0.045	0.032	0.046	0.151	0.046	0.686	0.082	0.092
2015	0.385	0.104	0.037	0.034	0.04	0.046	0.038	0.419	0.044	0.146
2018	0.313	0.097	0.032	0.037	0.031	0.052	0.032	0.330	0.038	0.150

From the results of sensitivity analysis, it can be seen that although the sensitivity of urban ecosystem health to changes of various indicators is decreasing in Zibo, there are still obvious differences. Based on the sensitivity index, the indicators can be divided into two categories, namely sensitivity indictor and non-sensitivity indictor. Since 2015, the sensitivity indexes of the proportion of tertiary industry in GDP (*x*_*4*_), the proportion of high-tech industry in GDP (*x*_*5*_), the registered urban unemployment rate (*x*_*6*_), urban sewage treatment rate (*x*_*9*_), comprehensive utilization rate of industrial solid waste (*x*_*10*_), harmless treatment rate of domestic waste (*x*_*11*_) and the Engel’s coefficient of urban residents (*x*_*18*_) are all greater than 0.2, obviously higher than other indicators. Changes of these indicators have a relatively large impact on urban ecosystem health, hence they are designated as sensitivity indicators. Other indicators with lower sensitivity index have a weak impact on urban ecosystem health, and are designated as non-sensitive indicators. Among the sensitivity indicators, except the Engel’s coefficient of urban residents (*x*_*18*_) which reflects the living standard of urban residents, the other six indicators reflect the structure and resilience of urban ecosystem, indicating that the structure adjustment and resilience change of ecosystem have a prominent impact on urban ecosystem health. According to the characteristics of industrial city, Zibo should strengthen urban ecological construction, improve the comprehensive waste treatment capacity, and at the same time continue to optimize the industrial structure, introduce high-tech industries, increase the employment opportunities for residents to effectively promote urban ecosystem healthy.

## Discussion

From the perspectives of ecology and system theory, an important feature of urban ecosystem health is rational structure and perfect function^11^. In addition, human beings are an important component of urban ecosystem, and urban ecosystem should also provide services for human beings and maintain human health. In this study, the vigor-structure-resilience-function-population health assessment framework was constructed to comprehensively reflect the overall characteristics of the urban ecosystem from different dimensions. Referring to previous studies and based on the assessment framework, we screened and supplemented the indicators to construct a relatively complete assessment index system in this study. Some indictors were added, such as air quality indicator reflecting the characteristics of industrial cities, proportion of high-tech industries indicator reflecting the industrial structure, harmless treatment of domestic waste indicator and environmental protection investment indicator reflecting the self-regulation ability of urban ecosystem, and medical security indicator reflecting the medical health level of urban residents, which was the improvement of the index system. Based on the complexity of urban ecosystem, although the indicators have been screened and supplemented in this study, the index system still needs to be improved due to the limitations of data acquisition and quantification. In future research, more data sources and indicator types need to be considered to further improve the index system and improve the accuracy of assessment. Although the index system established in this study is not the most ideal, it can still provide references for city managers and decision-makers to maintain urban ecosystem health in Zibo. This study analyzed the influencing factors of urban ecosystem health from the aspects of obstacle degree and sensitivity, which can not only quantitatively reflect the influence degree of different factors, but also reflect the change trend of the influence degree of factors. This is conducive to identifying the key factors affecting the healthy development of urban ecosystems. To a certain extent, these research methods can make up for the shortcomings of existing research methods.

From 2006 to 2018, the health level of urban ecosystem continued to improve in Zibo, which was closely related to the policies of “energy conservation and emission reduction” and “transformation of Old-New-Driving-Forces”. As an industrial city, ecosystem structure and resilience are currently the main factors affecting urban ecosystem health in Zibo. In the future, policies will continue to play an important role in improving urban ecosystem health. Therefore, urban green development policies should be formulated to balance the relationship between economic development and environmental protection, so as to promote urban ecosystem health. Zibo should adopt environmental protection policies to strengthen urban ecological construction and enhance the resilience of urban ecosystem, such as formulating strict pollutant emission standards, formulating incentive policies for technological innovation, clean, regenerated energy utilization policies, and increasing environmental protection input. In the second place, Zibo should formulate industrial development policies and seek for a new economic development model. Developing the tertiary industry and increasing the proportion of high-tech industries will help Zibo optimize its industrial structure and promote regional economic development. In addition, the lower urban population density is an obstacle factor affecting urban ecosystem health in Zibo. Talents are important for urban development. Studies have shown that improving the quality of the population is conducive to promoting healthy urban development^21,26^. Zibo is not attractive enough for talents now. In order to promote urban development, Zibo should learn from the experiences of other cities and formulate talent introduction policies to attract all kinds of talents to settle down.

Due to the impact of COVID-19 broke out in 2019, some indicator data changed greatly. In order to avoid the impact of data anomalies on the research results, the study period was selected from 2006 to 2018. The epidemic will have a negative impact on socio-economic development, which in turn will affect urban ecosystem health. The change characteristics of urban ecosystem health under the impact of the epidemic will be further studied in future research.

## Conclusion

Assessment of urban ecosystem health can help to identify the health status and limiting factors in an urban ecosystem, which can provide references for governments to formulate urban management strategies. In this study, an index system was constructed based on the vigor-structure-resilience-service function-population health framework to assess the health level of urban ecosystem from 2006 to2018 in Zibo. Obstacle degree analysis and sensitivity analysis were used to quantitatively analyze the main obstacle factors and sensitivity factors affecting urban ecosystem health. The conclusions are as follows:

Under the guidance of the new industrialization concept and the policy of transformation of Old–New-Driving-Forces, the overall health level of urban ecosystem showed a rising trend from 2006 to 2018 by optimizing the industrial structure and strengthening urban ecological construction, especially ecosystem vigor, structure, resilience and population health were significantly improved. Although the service function was not significantly improved, it continued to maintain a high level.

From 2006 to 2018, the number of main obstacle factors affecting urban ecosystem health showed a decreasing trend. The poor structure and ecological environment quality were still the main constraints on urban ecosystem health.

The results of sensitivity analysis showed that from 2006 to 2018, except for environmental protection investment, the sensitivity index of other indicators showed a decreasing trend, but the sensitivity of indicators was still significantly different. Ecosystem health was more sensitive to changes in structure indicators and resilience indicators. The impact of changes in a single indicator on urban ecosystem health gradually decreased, which was the result of gradual improvement of urban ecosystem, and also indicated that focusing only on one aspect of urban development could not effectively improve level of urban ecosystem health in the future. Therefore, promoting the coordination of socio-economic development and ecological environmental protection is a realistic choice to ensure urban ecosystem health in Zibo.

## Data Availability

All the data for this study are available from the corresponding author on reasonable request.
